# Motor-related oscillatory activity in schizophrenia according to phase of illness and clinical symptom severity

**DOI:** 10.1016/j.nicl.2020.102524

**Published:** 2020-12-03

**Authors:** Lauren E. Gascoyne, Matthew J. Brookes, Mohanbabu Rathnaiah, Mohammad Zia Ul Haq Katshu, Loes Koelewijn, Gemma Williams, Jyothika Kumar, James T.R. Walters, Zelekha A. Seedat, Lena Palaniyappan, J.F. William Deakin, Krish D. Singh, Peter F. Liddle, Peter G. Morris

**Affiliations:** a Sir Peter Mansfield Imaging Centre, School of Physics and Astronomy, University of Nottingham, University Park, Nottingham NG7 2RD, United Kingdom; b Institute of Mental Health, University of Nottingham, Jubilee Campus, Nottingham NG7 2TU, United Kingdom; c Nottinghamshire Healthcare NHS Foundation Trust, Nottingham NG3 6AA, United Kingdom; d CUBRIC, School of Psychology, College of Biomedical and Life Sciences, Cardiff, Cardiff University CF24 4HQ, United Kingdom; e Division of Neuroscience and Experimental Psychology, University of Manchester, Oxford Rd, Manchester M13 9PL, United Kingdom; f Department of Psychiatry & Robarts Research Institute, University of Western Ontario & Lawson Health Research Institute, London ON, Canada; g MRC Centre for Neuropsychiatric Genetics and Genomics, Cardiff University, CF24 4HQ, United Kingdom

**Keywords:** Psychosis, Schizophrenia, Transient beta events, Post-movement beta rebound, Oscillatory bursts

## Abstract

Magnetoencephalography (MEG) measures magnetic fields generated by synchronised neural current flow and provides direct inference on brain electrophysiology and connectivity, with high spatial and temporal resolution. The movement-related beta decrease (MRBD) and the post-movement beta rebound (PMBR) are well-characterised effects in magnetoencephalography (MEG), with the latter having been shown to relate to long-range network integrity. Our previous work has shown that the PMBR is diminished (relative to controls) in a group of schizophrenia patients. However, little is known about how this effect might differ in patients at different stages of illness and degrees of clinical severity. Here, we extend our previous findings showing that the MEG derived PMBR abnormality in schizophrenia exists in 29 recent-onset and 35 established cases (i.e., chronic patients), compared to 42 control cases. In established cases, PMBR is negatively correlated with severity of disorganization symptoms. Further, using a hidden Markov model analysis, we show that transient pan-spectral oscillatory ”bursts”, which underlie the PMBR, differ between healthy controls and patients. Results corroborate that PMBR is associated with disorganization of mental activity in schizophrenia.

## Highlights

• Beta rebound (PMBR) is reduced in recent-onset and established schizophrenia cases
• In established cases, PMBR is negatively correlated with disorganisation symptoms
• Hidden Markov model shows different transient pan-spectral bursts underlying PMBR.

## 1 Introduction

During movement, the beta oscillatory power (13–30 Hz) in the sensorimotor cortex is reduced, a phenomenon known as the movement-related beta desynchronization (MRBD); this is followed by a rebound of beta power above baseline level after cessation of the movement, known as the post-movement beta rebound (PMBR) (Pfurtscheller et al., 1996). The functional role of PMBR remains a topic of debate. Engel & Fries (2010), proposed that it reflects resumption of neural processing maintaining the status quo, while more recent evidence indicates that the oscillatory bursts that underlie the PMBR have a more general role re-establishing ongoing long-range communication (Seedat et al., 2020). Although the peak of the PMBR activity takes place within the sensorimotor cortex, there is good evidence that the strength of the PMBR is related to the effectiveness of long-range connectivity throughout a wider network (Tewarie et al., 2019). Therefore, malfunction of PMBR might be associated with disordered long-range connectivity.

Impaired coordination of brain activity associated with abnormal electrophysiological oscillations plays a role in the generation of symptoms of psychotic illnesses including schizophrenia (Uhlhaas et al., 2013). We have previously reported reduced magnitude of PMBR in schizophrenia during a stable phase of illness (Robson et al., 2016). The degree of reduction was significantly correlated with a composite measure of severity of symptoms and disability. We subsequently demonstrated that the correlation was strongest with the disorganization symptom dimension (Rathnaiah et al., 2020). We have also demonstrated in healthy individuals that magnitude of PMBR is inversely correlated with the severity of schizotypy, a personality variant that is considered to lie on a continuum with the abnormalities of mental functions occurring in schizophrenia (Hunt et al., 2019). Furthermore, in that sample of healthy individuals, the degree of reduction in magnitude of PMBR was most strongly correlated with a dimension of schizotypy reflecting disorganization of mental activity characteristic of classical schizophrenia (Wuthrich & Bates, 2006). Since the times of Bleuler and Kraepelin, disorganization has been considered the fundamental feature of schizophrenia. Heritability of the disorganization dimension is likely to be highest among the three symptom dimensions of schizophrenia (Rietkerk et al., 2008). In particular, severity of disorganization in schizophrenia is associated with persisting cognitive deficits and deficits in role function (Liddle, 2019). PMBR is also reduced in other neuropsychiatric conditions including fronto-temporal dementia (Hughes et al., 2018), multiple sclerosis (Barratt et al., 2017) and autism (Gaetz et al., 2020) suggesting that it might reflect a brain process associated with persisting disability in diverse conditions.

Schizophrenia is heterogeneous in time course (Carpenter & Kirkpatrick, 1988) but typically it is an illness characterized by acute episodes superimposed upon a degree of persisting symptoms and disability. The pathological processes that underlie persistence remain uncertain. In a review of the investigations of treatment resistant illness, Gillespie et al., (2017) concluded that compared to treatment-responsive patients, treatment-resistant patients tend to exhibit glutamatergic abnormalities, a lack of dopaminergic abnormalities, and significant decreases in grey matter. The negative and disorganization symptoms that are associated with impaired cognition and role function in the stable phase of illness (Rathnaiah et al., 2020) appear to reflect different pathological processes from apparently similar symptoms in the acute phase of illness. For example, florid formal thought disorder responds to dopamine blocking medication (Johnstone et al., 1978), but more subtle disorganization of thought and speech persists despite antipsychotic treatment in the chronic phase of illness (Spohn et al., 1986). Similarly, negative symptoms in the acute phase respond moderately well to treatment (Rolls et al., 2017), but respond poorly to treatment in the chronic phase.

Patient recruitment criteria in this study were designed to help delineate the similarities and differences between the pathophysiology of persistent symptoms and that of acute transient symptoms. The criteria for inclusion in the established illness group ensured that the symptoms in the established cases were treatment resistant. In contrast, recent-onset cases were expected to have symptoms characteristic of the acute phase of illness. As approximately 30% of first episode cases suffer treatment resistant symptoms (Lally et al., 2016), the recent-onset sample was expected to have greater heterogeneity in the degree of tendency to persistence. Nonetheless, separate examination of the pathophysiological correlates of symptoms in the recent-onset cases and the established cases offers the prospect of delineating the similarities and differences of the pathological processes generating acute-phase symptoms and persistent symptoms.

In this study we aimed to confirm the reduction of PMBR previously reported in schizophrenia, and examined the question of whether this abnormality is observed in both recent-onset illness and well-established illness. In addition, we tested the hypothesis that the reduction in PMBR is correlated with overall severity of illness and with the severity of the disorganization syndrome.

Furthermore, to provide further evidence relevant to the hypothesis that reduction of PMBR in schizophrenia might reflect a disturbance of long range connectivity, we investigated the occurrence of transient bursts of oscillatory activity occurring in the PMBR time window following motor responses. Recent works (Sherman et al., 2016, Shin et al., 2017) have shown that, rather than the classical picture of a smooth “oscillation” whose amplitude changes with time, the beta rhythm is “formed” (in part) from the recurrence of discrete and punctate events; each event can be thought of as a very short (e.g. a few hundred milliseconds) burst of activity. These bursts occur with a characteristic probability, which is altered by a task. For example, during movement execution, the probability of bursts becomes lower; during the PMBR the probability becomes higher (Little et al., 2019, Seedat et al., 2020). This means that, when summed over large numbers of trials, bursts combine to give the impression of a smooth variation in oscillatory amplitude.

Using a technique that entailed identifying Hidden Markov States in the time course of the MEG signal recorded during a simple visuo-motor task, Seedat et al (2020) demonstrated that coincident bursts of transient oscillatory activity with a spectral peak in the beta band occur in time windows of high coherence (i.e. phase-locking) between brain regions. From an examination of the relationship between the patterns of Amplitude Envelope Correlations between brain regions and the pattern of burst co-incidence between regions, Seedat et al concluded that bursts play an important role in driving functional connectivity. Thus, if the reduction of PMBR occurring in schizophrenia reflects an abnormality of long-range connectivity, we would predict a reduction in beta bursts quantified by identifying the relevant hidden Markov state with a spectral peak in the beta band, in the PMBR window. In principle, reduced PMBR might reflect reduction of frequency of occurrence, duration or amplitude of the bursts. Therefore, we compared all three aspects of the coincident bursts in the PMBR window in the two patient groups with healthy control participants.

## 2 Materials and methods

### 2.1 Participants

The study presented here formed part of SPRING (The Study of Psychosis and the Role of Inflammation and GABA/Glutamate), a multimodal collaboration between the Universities of Manchester, Nottingham and Cardiff. MEG data, using identical scanners and protocols, were acquired at the University of Nottingham (Sir Peter Mansfield Imaging Centre) and Cardiff University (CUBRIC) sites. Inclusion criteria for all participants were: aged 18–55 years; ability to understand and willing to give written informed consent; English as first language or fluent. Exclusion criteria were: current use of any medication which may interfere with the study, in the opinion of the investigator (not including treatment for schizophrenia); clinically significant neurological disorder; history of head injury with loss of consciousness > 5 min; current harmful use of, or recent dependence on, psychoactive substances (excluding nicotine); contraindications for magnetic resonance imaging (MRI) or MEG (e.g., claustrophobia, pregnancy, ferrous metal implants); taken part within the previous month as a participant in a clinical trial that involved taking a drug, being paid an inconvenience allowance, or having an invasive procedure (e.g., venepuncture > 50 ml, endoscopy).

Patients were required to fulfil the current DSM IV criteria for schizophrenia, schizoaffective disorder, or schizophreniform disorder. Participants with schizophreniform disorder were followed up after six months and the DSM diagnosis of schizophrenia was established by collecting information from the case records. We recruited 40 patients diagnosed with recent-onset psychosis (<5 years since diagnosis, with antipsychotic drugs exposure either absent or minimal (<12 weeks)) and 40 patients with established psychosis (10 or more years since diagnosis; a minimum of 8 weeks of stable antipsychotic drug treatment). We further recruited 42 controls, matched for age and sex, as well as parental occupation as a measure of socio-economic background (NS-SEC; Krieger et al., 2003). In each group, half of the patients were recruited and scanned in Nottingham, and the other half in Cardiff. Hereafter the groups may be referred to as RO (recent-onset), ES (established patients) and CT (controls).

Due to the inevitable age differences between the recent-onset and established patients, 10 control participants were matched to each patient group per site. Controls were recruited through advertisement posters placed in public places. Additional exclusion criteria for controls were: a personal history of psychosis or a related disorder (as determined by the MINI-international neuropsychiatric interview v5.0.0 for DSM-IV (Sheehan et al., 1998)); current or recent (within 2 years) presence of depressive symptoms or treatment with antidepressant medication; first degree relative with a history of psychosis. Participants in Nottingham were further excluded from participation if they had a blood-borne virus, this was due to a subsequent 13-C magnetic resonance spectroscopy (MRS) scan not reported here. Informed consent was obtained and participants were paid an inconvenience allowance for their participation. All procedures were approved by the UK National Research Ethics Service.

### 2.2 Data acquisition

All participants underwent data acquisition using both MEG and MRI, in addition to extensive screening, which examined their history of medication, nicotine, alcohol and drug use, a battery of cognitive tasks, and a neuropsychiatric interview. Blood samples were also taken for analysis of genetics and cytokines (not presented here).

In Cardiff, all data for each participant were acquired in one day, with a morning of cognitive tests and interviews, then blood acquisition, followed by an afternoon of imaging procedures, in which the MEG data were first acquired, then the 3 T anatomical MRI (and a 3 T 1H MRS dataset which is not presented here).

In Nottingham, participants were first screened at the Queen’s Medical Centre, including blood acquisition (and an ECG measurement to ensure eligibility for an additional ^13^C MRS acquisition). Participants that were approved to take part were then booked in for scan days at the Sir Peter Mansfield Imaging Centre. During the first scan day, participants underwent cognitive testing, followed by a semi-structured clinical interview in the morning. The MEG, 7 T MRI and 1H 7 T MRS acquisitions then took place in the afternoon.

At both sites, whole-head MEG recordings were made using a 275-channel CTF (CTF MEG Inc. Coquitlam, Vancouver, Canada) axial gradiometer system at a 1200 Hz sampling rate. An additional 29 reference channels were recorded for noise cancellation purposes and the primary sensors were analysed as synthetic third-order gradiometers (Vrba & Robinson, 2001). In both MEG systems a small number of channels were turned off, due to broken super conducting quantum interference devices (SQUIDs), flux transformers, or an excessive sensor noise. Participants were seated upright in the magnetically shielded room with their head supported either on a chin rest or with padding in the MEG helmet. Prior to recording, three electromagnetic coils were attached to the participant’s head at the nasion and preauricular points. These coils were energised continuously throughout acquisition and a magnetic dipole fit used to track the location of the head, and consequently head movements, throughout the recording. In addition to MEG data, we also recorded both the horizontal and vertical electro-oculogram measurements (using electrodes placed on the temples, and above and below the left eye, respectively) as well as the electro-cardiogram (via electrodes placed on each wrist).

#### 2.2.1 Visuo-motor task

Participants performed two runs of a visuo-motor task containing 50 trials per run. In each trial, a square black-and-white square-wave grating (approximately 15 degree of visual angle, 3 cycles per degree) was presented on a mean-luminance grey background. The grating was positioned in the lower-left visual quadrant relative to a central red fixation dot. This stimulus was present for a jittered interval, between 1.5 s and 2 s, and was followed by an 8–8.5 s variable rest phase in which the grey screen and fixation dot remained present. Stimuli were generated in MATLAB® (The Mathworks, Inc.) using the Psychophysics Toolbox extensions (Brainard, 1997, Pelli, 1997, Kleiner et al., 2007), and were presented via back projection onto a screen located ~ 40 cm in front of the subject, using a 60 Hz data projector (CTF Sanyo projector system, and also PROPixx Lite VPX-PRO-5000A in Nottingham, and CTF Sanyo projector system in Cardiff)). The visuo-motor recording took approximately 9 min per run, yielding a total of 50 trials per participant, per run. Temporal markers detailing the start and end of each visual presentation were added to the MEG data via a trigger channel. Participants rested their right hand on a board with their index finger placed against a box with a small lever attached. They were asked to push the lever sideways with their index finger, making a single, strong, brisk abduction for approximately 1 s, immediately after the visual grating disappeared. This action provided a small amount of resistance which helped the participant to ensure they were making the movement correctly. The index finger response was recorded using electromyography electrodes placed on the skin above the first dorsal interosseus muscle. Note that for two patients and one control, only a single recording run could be acquired due to technical issues with the MEG scanner.

#### 2.2.2 MRI and coregistration

A structural MRI was acquired on either 3 T GE or Siemens scanners (Cardiff), or 7 T Philips scanner (Nottingham). To achieve MRI/MEG co-registration, each centre followed their routine procedures. In Cardiff, fiducial markers were placed at fixed distances from three anatomical landmarks identifiable in the subject’s anatomical MRI, and their locations were verified afterwards using high-resolution digital photographs. In Nottingham, the participant’s head was digitised using a 3D digitiser (Polhemus, Colchester, VT), relative to the fiducial markers. The resulting head shape was subsequently fitted to the equivalent head shape extracted from the MRI. For 3 patients and 2 control participants, an MRI scan could not be acquired due to a technical fault or claustrophobia. For these participants, we replaced the missing MRIs with a structural MRI of the participant with the best matching head size, based on the lowest overall difference in Pythagorean distances between the fiducial points.

### 2.3 MEG data analysis

#### 2.3.1 Pre processing

The data were anonymised for group. All data were down sampled to 600 Hz and epoched into a window spanning from −1.5 to 8 s, relative to the offset of the visual grating. Head motion was referenced relative to the mean head position of each run and calculated per trial. Trials containing head motion values exceeding 5 mm from the mean were excluded. In two cases in the Cardiff sample, the nasion marker shifted on the skin. In these cases, movement (translation only) was approximated based on the left and right pre-auricular coils. Synthetic third-order gradiometer noise cancellation was applied, and the data were bandpass filtered to 1–150 Hz, and any DC offset removed. A single experimenter visually inspected the data and removed any trials with large artefacts (for example due to eye blinks). We further excluded datasets with a final number of trials less than half the original data (a minimum of 25 trials per run).

The pre-processed data were analysed in FieldTrip (version 20161011; Oostenveld, Fries, Maris, & Schoffelen, 2011). The participant’s coregistered MRI was imported and segmented using FieldTrip’s default segmentation. For two participants’ MRIs, this failed. In these cases, we performed extraction of the brain volume using FSL’s Brain Extraction Tool (Smith, 2002) and imported the resulting brain mask into FieldTrip.

#### 2.3.2 Source analysis

MEG data were further filtered to the beta (13–30 Hz) band, and downsampled to 300 Hz. Spatial filtering was performed using an LCMV beamformer on a 5 mm grid, warped to MNI template space, using a single shell volume conductor model (Nolte, 2003) to compute the forward solution. The covariance matrix was constructed using all of the filtered epoched data. Noise-normalised source power estimates were obtained for movement-related beta decrease (MRBD; 0.2 to 1.2 s post-grating-offset), post-movement beta rebound (PMBR; 2 to 3 s post-grating-offset) and baseline period (7 s to 8 s post-grating-offset) separately (using common beamformer weights). We then calculated the percentage change of the projected noise-normalised power, at each voxel, between active (MRBD or PMBR) and control (rest) windows.

Using the resulting source images, coordinates of peak locations within the right pre- or post-central gyrus for right MRBD and left pre- or post-central gyrus for left MRBD and PMBR were obtained (with regions defined based upon the Automated Anatomical Labelling (AAL) atlas; Tzourio-Mazoyer et al., 2002). At these peak locations, the time–frequency characteristics of the response were more fully investigated using a ‘virtual sensor’ approach in which new beamformer weights were constructed, based on all of the epoched data in a broad (1–120 Hz) frequency band. We performed a time–frequency analysis of this virtual sensor timeseries (multitaper method using a dpss taper, 4–100 Hz, 4 Hz smoothing, full −1.5 to 8 s window). Both runs were combined. To obtain beta power and frequency within the peak location of interest, we performed a spectral analysis (multitaper, Hanning window, 0–100 Hz in steps of 0.5 Hz) and calculated the percentage change between stimulus and baseline windows.

#### 2.3.3 Transient bursting using a hidden Markov model (HMM)

In order to analyse the bursting patterns that underlie beta modulation, we employed a Time-Delay-Embedded (TDE) Hidden Markov Model (HMM). Prior to the HMM application, a broad band (1–48 Hz) virtual electrode time course was created in the previously identified peak motor region for each participant. Following this, a 3-state univariate TDE-HMM (as used by Seedat et al. (2020)) was applied. An HMM assumes that a series of mutually exclusive ‘hidden’ states governs the observed values within a voxel time course, such that each timepoint is associated with one of the hidden states. The sequence is assumed to be Markovian so that any single time point only depends on the time point immediately preceding it. An observation model links the HMM state to the observable value in the regional time course. For example, in its simplest form the model would describe each state by a different Gaussian distribution, from which the observed values can be extracted. The mean and standard deviation of each Gaussian would define each state. Here we used a slightly more complex formulation with time-delay embedding (Vidaurre et al., 2018) where each state is characterised by a different autocovariance pattern, defined over a specified time window (duration 230 ms). These autocovariance patterns contain the spectral information of the signal when that state is active. The model inference was undertaken using a variational Bayesian method. The output comprises time courses specifying the probability of a specific voxel existing in any one of three states. These time courses are binarised such that a single exclusive state is “active” for each time point. This is achieved by thresholding the probability time courses above two thirds for each state. For each voxel, a single state best characterising the pan-spectral bursts which modulate the beta signal was identified. (Further description of this bursting algorithm method can be found in Seedat et al. (2020)).

**Fig. 1:**
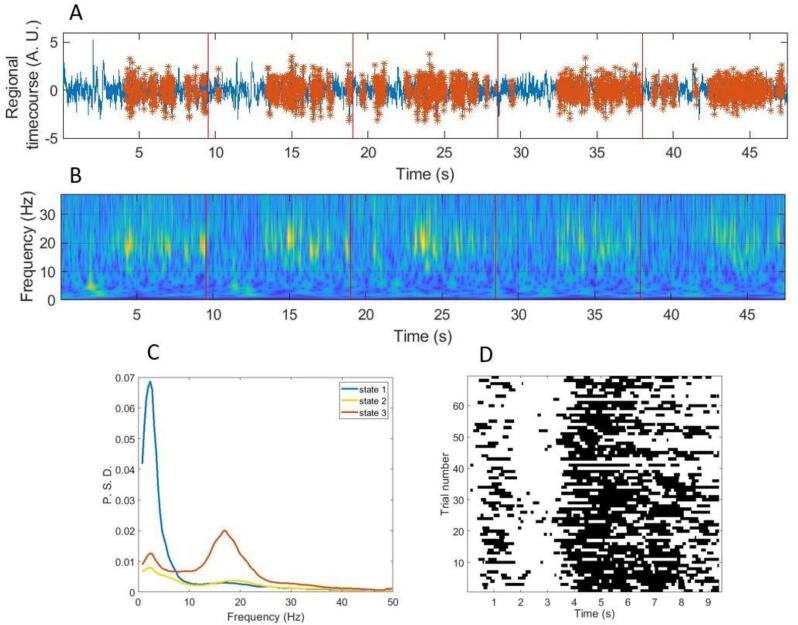
**HMM output for a single virtual sensor timeseries, in one representative healthy control participant.** A) Broadband (1–48 Hz) regional timecourse data (arbitrary amplitude) with beta burst state identified by the HMM (in red, with trials demarcated with red lines). B) Time-frequency decomposition of the same data generated using a Morlet wavelet transform (trials demarcated in red). C) Spectra showing the component frequencies of the three states identified from the HMM. The state most highly correlated with the beta envelope (the beta burst state 3) is shown in red. D) Raster-plot showing the binarised occurrences of the burst state for all trials, note how the MRBD and PMBR time periods can be easily distinguished. (For interpretation of the references to colour in this figure legend, the reader is referred to the web version of this article.)

A schematic of this process is shown in Fig. 1. Fig. 1A shows the 1–48 Hz filtered voxel time course, with occurrences of the burst state shown overlaid in red. Fig. 1B, for comparison, shows a wavelet-based time frequency decomposition of the same data. Data are shown across 5 trials of the visuomotor task, in an individual subject, with the voxel placed in the motor cortex. Note that periods of bursting correspond to high levels of beta activity in the time frequency representation. Fig. 1C shows the spectra associated with each of the three identified states; the burst state (state 3) is shown in red with a high contribution to beta activity. Finally, Fig. 1D shows a raster-plot of the occurrences of the burst state over trials, for a single subject; note the state probability declines during the MRBD window and increases during the PMBR window. These results are in line with previous reports using similar methodologies (Little et al., 2019, Seedat et al., 2020).

To quantify the burst parameters, we employed an established approach (Quinn et al., 2019, Heideman et al., 2020). Specifically, for each burst, we measured its amplitude (defined as maximum beta amplitude within the burst) and its duration (defined as the number of sequential timepoints that the HMM classified as existing in the burst state). The binarised state time courses were then reshaped into a matrix of time-points by trials (similar to Fig. 1D), and, summing across trials for each time point, we measured:

1) **Burst probability** – i.e. the probability of a burst occurring at that particular time point within the trial (i.e. number of occurrences of that time point with a burst, divided by the total number of trials (across all participants)).
2) **Burst amplitude** – i.e. the average amplitude of bursts which are found to occur at that specific point in time.
3) **Burst duration** – the average duration of bursts occurring at that time point.

In all three cases, this analysis gave us a time course showing how the burst parameter evolves across the average trial. These parametric time courses were plotted along with standard error across individual participants, within all three subject groups.

#### 2.3.4 Clinical assessment

Symptoms were assessed using the Positive and Negative Symptoms Scale (PANSS) (Kay et al., 1988). Scores for each of the three characteristic syndromes of schizophrenia: disorganization, psychomotor poverty (impoverishment), reality distortion (Liddle, 1987, Liddle, 2019) were determined by summing items loading on the relevant factors identified in the *meta*-analysis of the PANSS factor structure (Shafer & Dazzi, 2019). Details of the symptom items constituting these three factors are included in the Supplement.

#### 2.3.5 Assessment of exposure to medication (patients only)

Prescription of antipsychotic medication over the course of the illness was ascertained by examination of case files. The life-time exposure was quantified according to operational criteria on a scale from 0: No antipsychotic exposure, to 10: >10 years total exposure including high dose exposure of>5 years in duration (See Supplement for further details).

#### 2.3.6 Statistical analysis

We determined whether or not patients differed from controls in the peak source localizations of the MRBD and PMBR responses. To investigate whether there was a difference in the variance in any direction (X,Y and Z) between the groups, we used a multivariate ANCOVA including each component of the response: left and right MRBD and left PMBR, controlling for sex, site and age.

For the traditional power analysis, the dependent variable of interest is the percentage change from baseline to PMBR for the three groups. To initially test for a group difference in the dependent variables, we conducted a univariate ANCOVA, which allowed us to control for the covariates of age, site, sex and number of trials. For each ANOVA, we conducted *post hoc* tests and used a False Discovery Rate (FDR) approach to controlling for Type I errors arising from multiple comparisons. We used the Benjamini & Hochberg (1995) procedure to compute *q* values (“adjusted *p* values”) representing the probability of a false positive for each *post hoc* test. We set the criterion for significance at FDR < 0.05.

A set of linear regressions was performed with PMBR as the dependent variable and symptom severity as the predictor variable of interest (for total severity of PANSS and severity of each of the three characteristic syndromes of schizophrenia: disorganization, impoverishment, reality distortion). Age, sex, site and antipsychotic medication exposure were treated as covariates (testing was repeated with age removed as a covariate). Separate regression models were estimated for recent-onset cases and for established cases, as well as for the whole patient group.

For the HMM bursting algorithm, we investigated the behaviour of three bursting characteristics: burst count, burst duration and burst amplitude. For each characteristic, we took an average of values from the baseline and PMBR windows and calculated the percentage change from baseline to PMBR. We then performed a univariate ANCOVA on the percentage change values for the three groups to initially look for any group differences. Each ANCOVA controlled for age, site and sex. Following this, FDR-corrected post-hoc tests as described above were carried out between each group to identify any group pairs that differed in their values.

All statistical analyses were undertaken using IBM SPSS Statistics 25.

## 3 Results

### 3.1 Participants

A number of recruited participants were excluded from the analysis. From the recent-onset group, 1 withdrew from the study, 2 did not undergo the visuo-motor task on the day, 1 dataset could not be used due to technical difficulties with head localisation, and a further 7 were excluded after pre-processing, due to noisy data, excessive head movement and/or poor abductions. From the established patient group, 1 was excluded due to MEG/MRI contraindications, 1 withdrew from the study, and a further 3 were excluded after pre-processing. Following pre-processing, the mean and standard deviation of trials per group are as follows: recent-onset patients 84.8 (20.9), established patients 82.7 (19.5), and controls 88.6 (17.7). A one-way ANOVA showed that there were no group differences in the mean number of trials used in further analysis, F(2) = 0.912, p = .405.

A total number of 29 recent-onset patients (mean age 23.7 (SD 5.8), 6F), 35 established patients (mean age 40.3 (SD 7.6), 9F) and 42 control participants (mean age 32.2 (SD 9.9), 12F) had data suitable for further analysis. A clinical demographic table with further details of the participants removed during pre-preprocessing and the remaining participants taken forward for analysis can be found in the Supplement.

### 3.2 Source localisation and time course for visuo-motor task

**Fig. 2:**
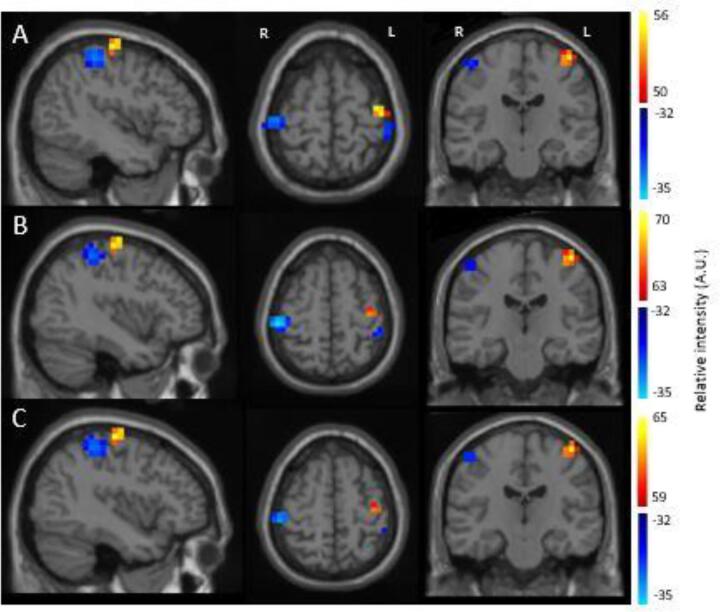
**Visuo-motor task source localisation.** Localisations shown for beta desynchronization (blue) and beta rebound (yellow/red) (radiological view; left on right) for A) CT group, B) RO patients and C) ES patients. Images were thresholded to show > 90% of maximum intensity for PMBR and minimum intensity for MRBD. (For interpretation of the references to colour in this figure legend, the reader is referred to the web version of this article.)

The bilateral movement-related beta desynchronization (blue) and contralateral post-movement beta rebound (yellow/orange) were localised independently in relation to baseline (Fig. 2). The MRBD was localised separately for the left and right hemispheres, with the peak found within the AAL masked regions of pre- and post-central gyri. The PMBR component is primarily a contralateral response and therefore was localised within the left AAL pre- and post-central gyri. Localisations are consistent with previous literature, i.e. the beta desynchronization is bilateral and posterior to the beta rebound, which occurs contralateral to movement (Barratt et al., 2017, Fry et al., 2016, Jurkiewicz et al., 2006). Table 1 shows the mean and standard deviation MNI values for the peak localisations, shown in cm.

**Table 1:** Mean and standard deviation of MNI peak locations (cm) in controls and patients for the three separate response components.

	Right MRBD			Left MRBD			Left PMBR		
	X	Y	Z	X	Y	Z	X	Y	Z
Controls	4.63 (0.8)	−2.53 (0.75)	5.29 (0.96)	−4.34 (0.97)	−2.46 (1.15)	5.55 (1.17)	−3.85 (0.79)	−1.96 (0.75)	5.94 (0.87)
RO patients	4.25 (0.83)	−2.36 (0.86)	5.68 (0.89)	−3.89 (0.87)	−2.86 (1)	5.79 (1.08)	−3.79 (0.86)	−2.18 (0.79)	5.89 (0.95)
ES patients	4.24 (0.93)	−2.63 (0.96)	5.56 (0.95)	−4.19 (0.78)	−3 (0.85)	5.69 (0.74)	−3.74 (0.91)	−2.12 (0.92)	5.99 (0.92)

We statistically compared the peak source localisations for the two patient groups and controls. A multivariate ANCOVA was conducted comparing the three MNI co-ordinates: X, Y and Z, from each component of the response: left and right MRBD and left PMBR for each group, controlling for sex and site. There were no statistically significant differences between the three groups, F(18,186) = 1.16, p = .29, Wilks’ Ʌ = 0.81, partial ƞ^2^ = 0.1.

**Fig. 3:**
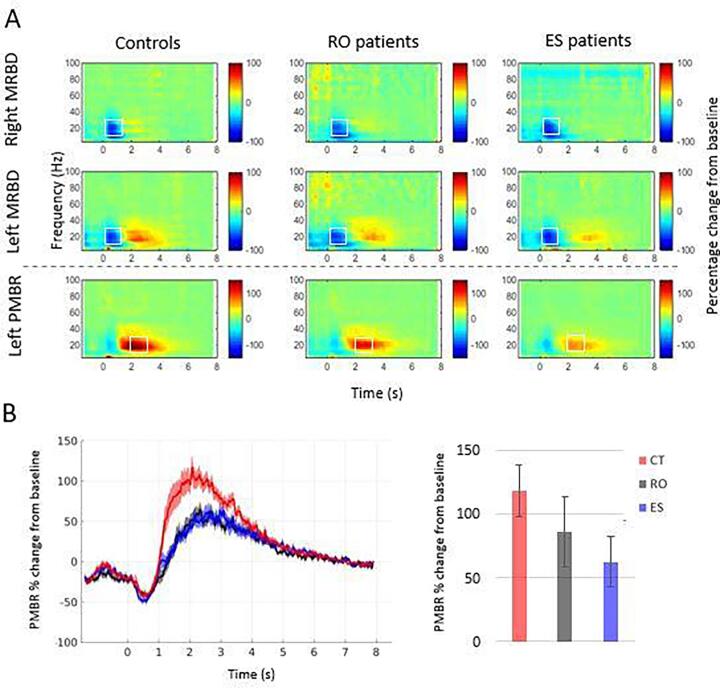
**Visuo-motor source power results** A) Scale shows power relative to baseline. Top line shows the right MRBD, middle line shows the left MRBD and bottom line shows the left PMBR for controls, recent-onset (RO) and established (ES) patients. B) Left: A representation of the beta frequency (13–30 Hz) time course for the controls, recent-onset patients and established patients separately, showing percentage change of the PMBR from baseline. Mean and SEM shown. Right: Bar chart of the difference between baseline and PMBR windows as a percentage change. Mean and CI shown. Graphs do not include outliers.

Fig. 3 shows the time–frequency representations of all three regions of interest, for all groups. Outliers were identified for the left and right MRBD variables and the left PMBR variable for each group using the standardized residuals derived from a univariate analysis of variance of the dependent variable. Any data point that had a standardized residual value > 3 was removed. Data were then checked for any interaction effect between variables, homogeneity of variance using Levene’s test, and heteroscedasticity using White’s test, these are not reported if found non-significant. Symptom severity (described in section 3.4 below) was the only statistically significant potential confound that differed between the Cardiff and Nottingham groups. Therefore for all other analyses, we report the results of statistical tests in which site was not included in the model. There is likely to be a confound of age due to the nature of the groups being compared, however covarying this information out of the statistical comparison could lead to Type II error. For this reason, we are using tests that both include and do not include age as a covariate, the full results of the latter can be found in the Supplement. Where results were different when age is included or excluded as a covariate, we performed a further hierarchical regression analysis to ascertain the contribution of age in that instance.

The MRBD response could be clearly visualised during the 0–2 s window in the top two rows of spectrograms, localised specifically to the peak MRBD. On visual inspection there did not appear to be any difference in the size of the MRBD between the groups for either the left or right hemispheres. This was confirmed statistically by a one-way univariate ANCOVA comparing the MRBD values averaged across participant groups, averaged across the beta frequency band and also averaged over the 0.2–1.2 s time window as per Robson et al. (2016). Prior to running the statistical model, we ensured that there were no significant interactions between any variables. The statistical model only included main effects with no interactions, and controlled for sex as a fixed factor, and age and number of trials as covariates. For the left MRBD, one female established patient outlier was removed.

There was no main effect of group for the left hemisphere MRBD: F(2,99) = 2.02, p = .139, however in this case White’s test for heteroscedasticity was significant (ꭓ^2^ (16) = 28.2, p = .03). There was no main effect of group for the right hemisphere MRBD: F(2,100) = 2.09, p = .128.

Repeating the test with removal of age as a covariate resulted in a different pattern of results for both the left and right MRBD. For both components, there was a significant effect of group, reflecting a significant reduction in MRBD in ES cases relative to controls, in both left and right hemispheres. However hierarchical regression revealed that age accounts for this effect (see Supplement).

The PMBR could clearly be seen in the left hemisphere in the control participants occurring within the 2–3 s window. The same effect could be seen in the patient groups, however it appeared much diminished, particularly in the established patients. Outlier inspection led to removal of one female recent-onset patient and one female established patient from further statistical analysis. Statistical univariate analysis of covariance of the PMBR values, averaged across participants per group, across the beta frequency band and within the 2–3 s window, controlled for age, sex and number of trials, show a significant main effect of group: F(2,98) = 11.9, p < .001.

Post-hoc tests of the estimated marginal means showed that the CT group had a significantly higher percentage change of PMBR (116.92 ± 8.96) than ES patients (53.95 ± 11.26, q < 0.001) as well as a significantly higher percentage change than the RO patient group (77.07 ± 12.49, q = 0.011). There was no significant difference between the ES and RO patient groups (q = 0.195).

### 3.3 Relationship between beta effects and abduction latency

We investigated the possibility that task-related behavioural differences between the groups might contribute to the observed beta effects. ANOVA revealed no significant main effect of group on the average abduction latency of each participant: F(2,101) = 1.26, p = .288. To further envisage the relationship between abduction latency and magnitude of beta oscillatory effects, we visually inspected scatter plots for each beta variable (see Supplement for scatter plots). No relationships were observed, therefore no further statistical analysis was undertaken.

### 3.4 Relationship between PMBR and clinical variables

Clinical symptom severity scales used in further analysis were examined for site confounds using non-parametric t-tests. Significant site differences were evident for Total PANSS (Z = -2.42, p = .015) and reality distortion scales (Z = -2.62, p = .009), therefore we controlled for site in further analysis. We firstly examined whether there were any significant relationships between the series of symptom severity scales and patient group by performing an ANCOVA for each scale controlling for antipsychotic medications, sex, site and age (tests also repeated without controlling for age). There were no significant differences in symptom severity between RO and ES groups. Further details can be found in the Supplement.

**Fig. 4:**
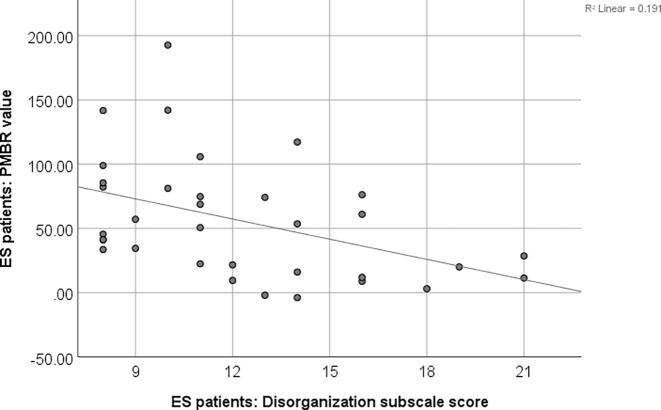
**PMBR relationship with PANSS.** The plot shows the disorganization PANSS subscale score factor correlated with the PMBR percentage change from baseline for the established patient group.

We performed a series of two-tailed Pearson’s partial correlation comparing the patient group PMBR scores with Total PANSS score, disorganization factor, impoverishment factor and reality distortion factor, controlling for age, sex, site and antipsychotic medication exposure score. Only significant correlations are reported here (Fig. 4), the remaining correlations and their associated graphs can be found in the Supplement. PANSS disorganization factor was found to be strongly correlated with PMBR in ES patients; r(28) = -0.42, p = .02. This relationship still holds when age is removed as a covariate: r(29) = -0.42, p = .02.

**Fig. 5:**
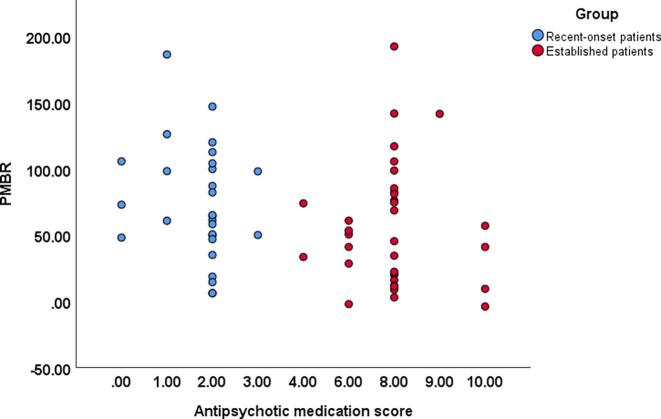
**PMBR relationship with antipsychotic exposure score.** The plot shows the relationship between the PMBR and antipsychotic medication score for recent-onset (RO; blue) and established patients (ES; red). (For interpretation of the references to colour in this figure legend, the reader is referred to the web version of this article.)

A Pearson’s two-way partial correlation controlling for age, sex and site was performed on each patient group, comparing the PMBR and the antipsychotic medication exposure score (Fig. 5). Neither correlation was significant, for the RO group r(23) = -0.069, p = .744, and for the ES group r(29) = 0.158, p = .397.

### 3.5 Group differences in transient burst events

**Fig. 6:**
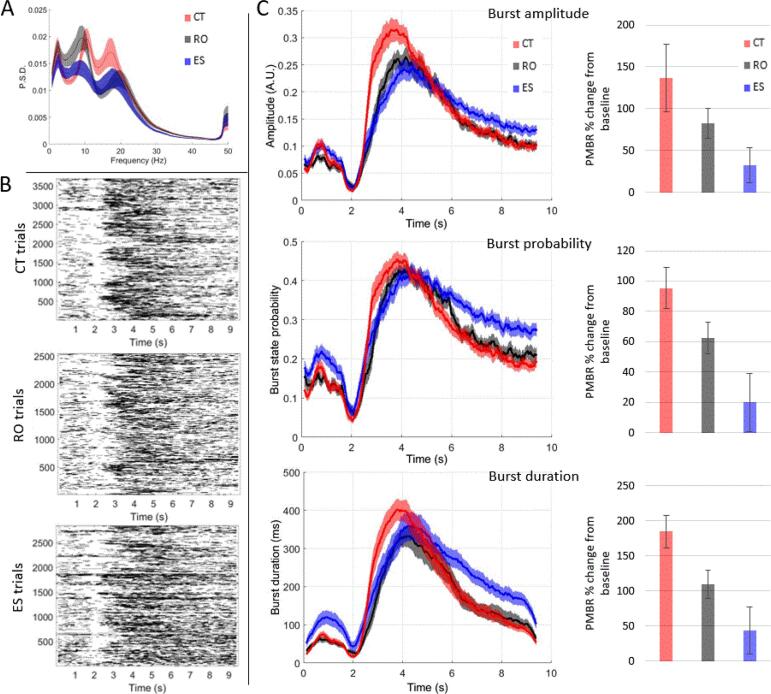
**Characteristics of HMM identified task-related bursts.** A) Power spectral density of chosen burst state, red for controls (CT), black for recent-onset patients (RO) and blue for established patients (ES). Mean and SEM shown. B) Raster plots of binarised bursts across time per trial. Top plot shows controls, middle plot shows recent-onset patients, bottom plot shows established patients. C) First column: time courses of HMM burst characteristics, top to bottom, amplitude, burst state probability and burst duration. Mean and SEM shown. Second column: Percentage change of the relevant HMM burst characteristic within the PMBR (2.5–4.5 s) relative to the baseline window (6–9 s) for each time course. Mean and CI shown. (For interpretation of the references to colour in this figure legend, the reader is referred to the web version of this article.)

Fig. 6 shows group differences in the task-related burst characteristics. Fig. 6A shows the power spectral density of the burst state by group, Fig. 6B shows representations of binarised bursts for the control and two patient groups separately and Fig. 6C shows the time courses of the burst characteristics and the percentage change from baseline for each characteristic.

Outliers for the MEG data were identified by first examining the standardized residuals of each variable for each group using a univariate analysis of variance. Any data point with a standardized residual > 3 was removed. This resulted in removal of one recent-onset patient and one established patient from all three burst characteristic variables. Data were then checked for any interaction effect between variables, homogeneity of variance using Levene’s test, and heteroscedasticity using White’s test. In all cases the Levene’s and White’s tests were found to be significant, therefore all burst characteristic variables were subsequently log-transformed, and checked that they henceforth complied with these assumptions. There were no significant differences between sites in the burst characteristics, so site was not included in the reported analyses. Comparison of the post-movement window percent change from baseline across the three groups was conducted using a univariate ANCOVA for each burst characteristic, controlling for sex with and without age and looking for a main effect of group with no interactions.

For burst amplitude, there was a significant main effect of group, F(2,73) = 6.05, p = .004. Follow-up post-hoc tests on the estimated marginal means showed that there was a significant difference in burst amplitude percentage change from baseline between the CT (4.77 ± 0.21) and ES (3.65 ± 0.29, q = 0.006) group but not between the CT and RO (4.15 ± 0.29; q = 0.117) groups, nor between the two patient groups (q = 0.261). A similar pattern of results was seen after removal of age as a covariate (see Supplement for full results).

For burst count, there was a significant main effect of group on post-movement percentage change from baseline, F(2, 59) = 10.49, p < .001. Follow-up post-hoc tests showed that there was a significant difference in post-movement percentage change from baseline between the CT (3.87 ± 0.25) and ES (2.39 ± 0.39, q = 0.003) groups, as well as between the CT and RO (2.39 ± 0.34, q = 0.003) groups, but no difference between patient groups (q = 0.995). A similar pattern of results was seen after removal of age as a covariate.

For burst duration, there was a significant main effect of group on post-movement percentage change from baseline, F(2,74) = 4.48, p = .02. Follow-up post-hoc tests of the estimated marginal means showed that there was a significant difference in post-movement percentage change from baseline between the CT (3.58 ± 0.18) and ES group (2.69 ± 0.25, q = 0.012), but not between the CT and RO (3.28 ± 0.25, q = 0.341) groups, nor between the patient groups (q = 0.189). A similar pattern of results was seen after removal of age as a covariate.

## 4 Discussion

Our findings confirmed that PMBR is reduced in schizophrenia and demonstrated that the reduction occurs in both the recent-onset and established phases. MRBD was also diminished in the established group relative to controls but this effect was not significant after controlling for the effect of age. Furthermore, our analysis of oscillatory bursts with a spectral peak in the beta band confirmed and extended the PMBR findings. In accord with the expectation that the time course of the beta signal observed in trial-averaged data reflects the superposition of discrete brief burst of oscillatory activity during individual trials, we observed a substantial increase in the amplitude; frequency of occurrence; and duration of bursts in the PMBR window relative to the baseline period in all three groups. In the case of frequency of occurrence of bursts, this increase of bursts in the PMBR window was significantly less in both recent-onset and established cases, relative to controls. There was no significant difference in burst rate between the two patient groups. In the case of both amplitude and duration of bursts in the PMBR window, there was a significantly smaller increase in established cases, relative to that in healthy controls. The recent-onset cases did not exhibit a significantly smaller increase in amplitude or duration of bursts in the PMBR window relative to healthy controls, though again, for these two measures, there were no significant difference between the two patient groups.

The overall pattern of differences between groups in the oscillatory effects of interest did not reveal any statistically significant differences between the two patient groups. Nonetheless, there was some evidence indicating a greater degree of abnormality in the established cases than the recent-onset cases. In particular, the increases in both amplitude and duration of oscillatory burst in the PMBR window relative to baseline were significantly smaller in established cases than in controls whereas neither of these measures of beta activity in the PMBR window were significantly smaller in the recent-onset cases than in controls. Furthermore in the bar charts in Fig. 3B and Fig. 6C illustrating the magnitude of the oscillatory effects of interest in the three groups, for all four measures the recent-onset patients exhibit effects intermediate between those of healthy controls and established cases. Overall, the evidence indicates a lesser magnitude of the reductions of the PMBR-related effects relative to controls in the recent-onset cases.

In light of the conclusion by Seedat et al (2020), that oscillatory bursts play an important role in driving functional connectivity as assessed by oscillatory Amplitude Envelope Correlations between brain regions, our observation of reduction of the bursts in the PMBR window in schizophrenia, especially in the established cases, is consistent with the hypothesis that reduced PMBR in schizophrenia reflects an abnormality of long range connectivity.

In the established phase, reduction in PMBR was correlated with the severity of disorganization as measured by the PANSS factor. This is consistent with our previous finding (Rathnaiah et al, 2020) that disorganization symptoms were the symptoms exhibiting the strongest negative correlation with PMBR in a sample of established cases of schizophrenia in a stable phase of illness. Insofar as reduced PMBR reflects an abnormality of long range connectivity, our findings suggest that persistent disorganization is associated with a disturbance of long range connectivity.

Correlations with severity of symptoms in the recent-onset phase of illness were not significant. It should be noted that due to the inclusion criteria, the recent-onset group included cases that were still actively psychotic as indicated by substantial scores for reality distortion symptoms. Mean severity of symptoms was similar in recent-onset cases and established illness, but it is noteworthy that several recent-onset cases had high PANSS total scores, reflecting continuing active psychosis. The partial response of florid formal thought disorder to dopamine blockade during an acute episode (Johnstone 1978) suggests that different pathophysiological processes contribute to disorganization in acute and stable phases. Our findings indicate that only the pathophysiological process characteristic of persistent disorganization is associated with reduced PMBR. Our findings do not preclude the possibility that this pathophysiological process might occur in those recent-onset cases predisposed to continuing disorganization, but its influence might be masked by more florid acute pathophysiology.

Insofar as disorganization during the prodromal phase (Ziermans et al., 2014) and also during the stable phase of established illness (Liddle, 1987, Liddle, 2019) is associated with persisting impairment of occupational and social function, our finding of an association between reduced PMBR and persistent disorganization does support the hypothesis that reduced PMBR is associated with risk of persisting disability. However our finding of reduced PMBR in recent-onset cases neither supports nor dis-confirms the hypothesis that it is a specific marker for risk of persisting disability.

It is noteworthy that Seedat et al (2020) demonstrated in healthy participants that the pattern of coincident bursts with a spectral peak in the beta band were associated with the pattern of functional connectivity predominantly in posterior brain regions. In light of the observation by Shin et al (2017) that beta bursts decrease the probability of detection of visual stimuli presented near the level of threshold for detection, it is plausible that beta bursts play an important role in the balance between endogenous and exogenous influences on the allocation of brain processing resources. Reduction of beta bursts might reflect a shift of this balance in favour of exogenous processing at the expense of endogenous processing. In light of the evidence suggesting that PMBR reflects confirmation of the ‘forward model’ guiding motor acts (Cao & Hu, 2016) the observed decrease in PMBR is schizophrenia is consistent with a decrease in endogenous control of behaviour. Such a shift in the balance between endogenous and exogenous processing in schizophrenia is consistent with the findings of excessive posterior functional connectivity in schizophrenia (but not in psychotic bipolar disorder) during visual working memory performance, assessed using fMRI (Palaniyappan & Liddle, 2014). Palaniyappan and Liddle suggested that this might reflect inefficient cortical processing. The implications of abnormal functional connectivity measured using fMRI with that measured using MEG are debatable, as inefficient neural recruitment would be expected to be associated with increased local BOLD signal and potentially with increased functional connectivity between regions, whereas the regional MEG signal is determined by locally coherent activity and might decrease if neural recruitment is inefficient. Thus PMBR potentially provides complementary information to that provided by fMRI. Further investigation of the relationship between MEG and fMRI measures of connectivity is warranted.

We observed that the reduction of PMBR is not correlated with exposure to antipsychotic medication. It is noteworthy that the observation of the association of diminished PMBR with schizotypy, particularly in those with more marked disorganization features, in an un-medicated non-clinical sample (Hunt et al., 2019), further indicates that the diminution of PMBR in disorders in the schizophrenia spectrum can arise independently of antipsychotic medication.

Bardouille & Bailey, (2019) have recently shown that there is a correlation between the age of healthy volunteers and PMBR, with older participants having a smaller response. In their study, Bardouille & Bailey (2019) examined a very large cohort of healthy volunteers in an online database and found a significant effect of age. This is a relevant consideration in the context of our study, as the established group of patients in general were older than the recent-onset group, however, both when controlling for age and not controlling for age, both patient groups had significantly smaller PMBR than the control participants. Further, there was no significant difference between the recent-onset and established groups in the trial averaged PMBR amplitude.

To conclude, our study has shown that the PMBR is reduced in established and recent-onset patients when compared to controls. The separate analysis of different stages of the illness extends our understanding of the likely role of the impairment of processes generating PMBR in the pathophysiology of schizophrenia, and raises new questions. We have confirmed that PMBR is associated with the disorganization of mental activity in the stable phase of illness. Furthermore, our demonstration that rate, amplitude and duration of bursts in the PMBR window are reduced in the stable phase reveals a comprehensive relationship between the mechanism of generation of beta bursts and the risk of persisting disorganization. In light of the conclusion by Seedat et al (2020) that oscillatory bursts play an important role in driving functional connectivity, together with the evidence that persisting disorganization is associated with poor functional outcome in schizophrenia, our findings open an avenue for further investigation of the mechanisms underlying both persisting disorganization and risk of poor functional outcome in schizophrenia. Our demonstration of reduced PMBR in recent-onset cases raises the question of whether or not reduced PMBR indicates a risk of poor functional outcome in some of these cases. Our observation of a lack of correlation with disorganization was possibly due to the confounding effects of acute disorganization. A longitudinal study of the clinical correlates of PMBR in the early phase of psychotic illness is warranted.

## CRediT authorship contribution statement

**Lauren E. Gascoyne:** Conceptualization, Software, Formal analysis, Investigation, Data curation, Writing - original draft, Visualization. **Matthew J. Brookes:** Conceptualization, Software, Resources, Methodology, Writing - original draft. **Mohanbabu Rathnaiah:** Conceptualization, Formal analysis, Validation, Investigation, Data curation, Supervision, Writing - original draft, Project administration. **Mohammad Zia Ul Haq Katshu:** Conceptualization, Investigation, Supervision, Project administration, Funding acquisition. **Loes Koelewijn:** Conceptualization, Software, Formal analysis, Investigation, Data curation. **Gemma Williams:** Conceptualization, Formal analysis, Investigation, Data curation, Project administration. **Jyothika Kumar:** . **James T.R. Walters:** Conceptualization, Supervision, Funding acquisition. **Zelekha A. Seedat:** Methodology, Software. **Lena Palaniyappan:** Conceptualization, Supervision, Funding acquisition. **J.F. William Deakin:** Conceptualization, Project administration, Funding acquisition. **Krish D. Singh:** Conceptualization, Software, Formal analysis, Writing - original draft, Supervision, Project administration, Funding acquisition. **Peter F. Liddle:** Conceptualization, Formal analysis, Writing - original draft, Supervision, Visualization, Project administration, Funding acquisition. **Peter G. Morris:** Conceptualization, Validation, Resources, Writing - original draft, Supervision, Project administration, Funding acquisition.
