# Supplementary material for: Motor-related oscillatory activity in schizophrenia according to phase of illness and clinical symptom severity
Source: Neuroimage Clin. 2020 Dec 3;29:102524. doi: 10.1016/j.nicl.2020.102524 (PMC7750164; doi:10.1016/j.nicl.2020.102524)
Supplement: Supplementary data 1 [file mmc1.docx]

**Supplement**

**Clinical and demographic table for participants**

The following table shows in **black** the characteristics of the participants taken forward for final analysis, and in **red** the characteristics of patients excluded during pre-processing of the MEG task.

| Characteristics | RO *N=29*  *N=7* | ES *N=35*  *N=3* | CT *N=42* | |
| --- | --- | --- | --- | --- |
| Age (years; mean (std)) | 23.7 (5.8)  24.8 (3.6) | 40.3 (7.6)  32.6 (3.8) | 32.2 (9.9) | |
| Sex (M/F) | 23/6  4/3 | 26/9  3/0 | 30/12 |  |
| Handedness (Right/Left/Both) | 26/2/1  7/0/0 | 33/1/1  3/0/0 | 33/8/1 |  |
| Parental NS-SEC (mean (std)) | 2.6 (1.6)  2.9 (1.7) | 2.7 (1.8)  2.3 (2.3) | 2.1 (1.5) |  |
| Duration of illness (months; mean (std)) | 8.7 (10.8)  5.7 (2.1) | 183.3 (58.8)  162 (21.6) | - |  |
| Antipsychotic medication score (mean (std)) | 1.7 (.81)  2.3 (.49) | 7.7 (1.4)  8 (0) | - |  |
| Total PANSS (mean (std)) | 57.9 (16.8)  46.7 (10.5) | 52.3 (16.6)  50.3 (5.8) | 32.2 (3.7) |  |
| PANSS Disorganization subscale (mean (std)) | 12.3 (4.3)  9.6 (1.4) | 12.2 (3.9)  10.7 (2.5) | 8.6 (1.2) |  |
| PANSS Impoverishment subscale (mean (std)) | 13.1 (5.8)  12.4 (6.6) | 13.5 (8.2)  14.3 (6.8) | 7.1 (.26) |  |
| PANSS Reality Distortion subscale (mean (std)) | 13.2 (6.1)  8.7 (4.9) | 10.4 (4.4)  8.6 (3.2) | 5.3 (.92) |  |

**Scatter plots of average latency and beta values**

**
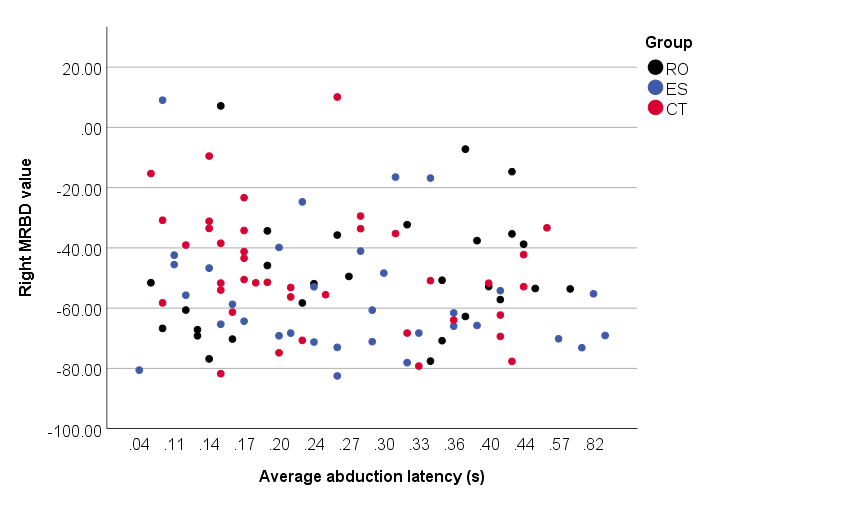
**

*
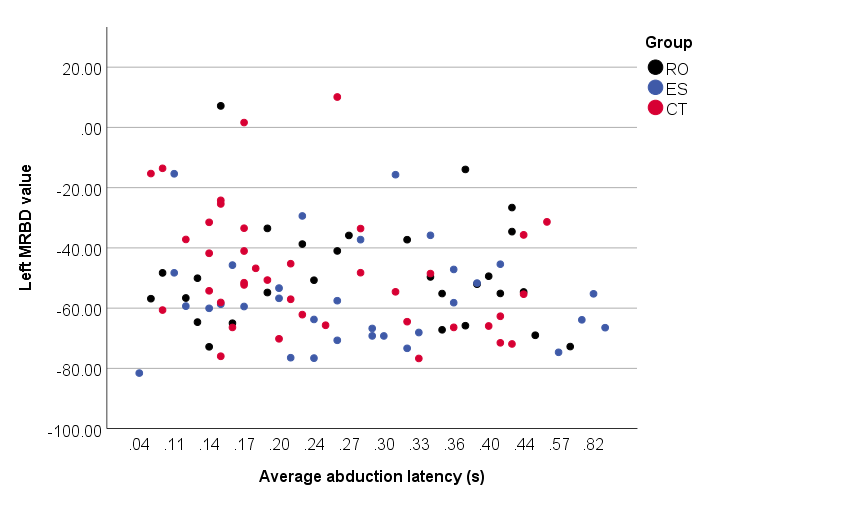
*


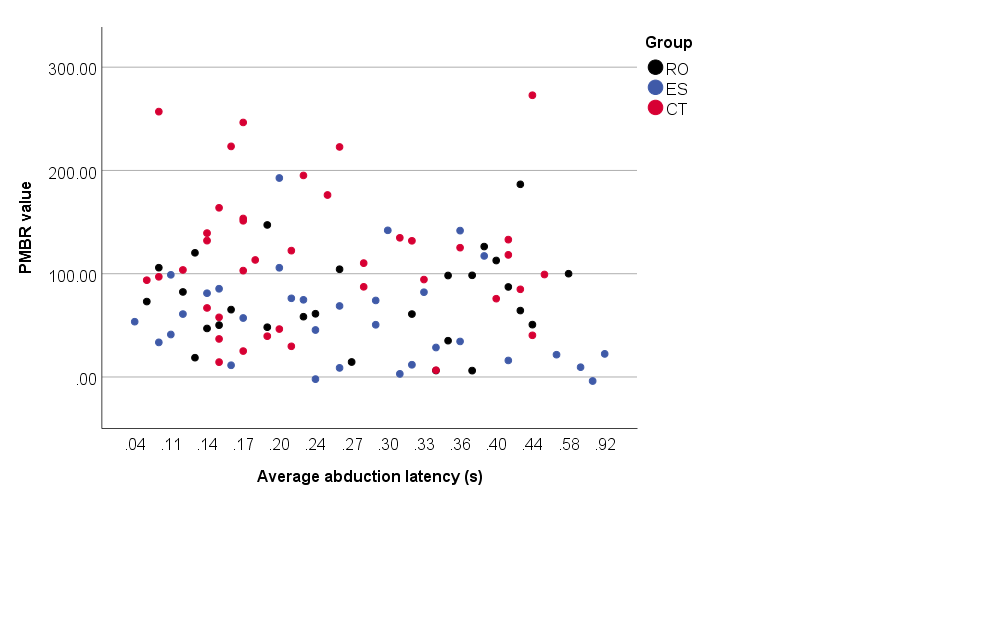


**PANSS Factor Information**

Based on the recent meta-analysis of PANSS items (Shafer & Dazzi, 2019), we used the five syndrome clusters/factors derived from PANSS as below:

| **Reality Distortion**  **(Positive symptoms)** | **Impoverishment (Negative symptoms)** | **Disorganization** | **Affect (Depression-Anxiety)** | **Resistance**  **(Excitement)** |
| --- | --- | --- | --- | --- |
| P1 Delusions | N2 Emotional withdrawal | P2 Conceptual disorganization | G2 Anxiety | P7 Hostility |
| G9 Unusual thought content | N1 Blunted affect | G11 Poor attention | G6 Depression | G14 Poor impulse control |
| P3 Hallucinatory behaviour | N4 Passive apathetic social withdrawal | N5 Difficulty in abstract thinking | G3 Guilt feelings | P4 Excitement |
| P6 Suspiciousness and persecution | N6 Lack of spontaneity | G13 Disturbance of volition | G4 Tension | G8 Uncooperativeness |
| P5 Grandiosity | N3 Poor rapport | N7 Stereotyped thinking | G1 Somatic concern |  |
|  | G7 Motor retardation | G5 Mannerisms/  posturing |  |  |
|  | G16 Active social avoidance | G15 Preoccupation |  |  |
|  |  | G10 Disorientation |  |  |

We assigned equal weights to all the individual symptoms within each factor/syndrome cluster. Scores were obtained by summing up the individual symptom item scores within each factor/syndrome. Within each syndrome, the allocated symptoms were weighted equally.

**Total antipsychotic exposure range**: range 0-10 based on duration and dose:

0: No antipsychotic exposure

1: Low dose exposure only (any duration)

2: Less than 1 year total exposure including some medium but no high dose exposure

3: Less than 1 year of total exposure including some high dose exposure

4: One to five years total exposure including medium but not high dose exposure lasting more than one month

5: One to five years total exposure including with some high dose exposure lasting more than one month.

6: Five to 10 years total exposure including medium dose but no high dose exposure lasting more than one month

7: Five to 10 years total exposure with some high dose exposure lasting more than one month

8: Greater than 10 years exposure including medium but no high dose exposure lasting more than one month

9: Greater than 10 years total exposure including with high dose exposure of between one month and 5 years in duration

10: Greater than 10 years total exposure including high dose exposure of greater than 5 years in duration

**Time-frequency power analysis results – without including age as a covariate**

*Left and right MRBD*

The left hemisphere MRBD shows a significant effect of group F(2,100) = 3.67, p = .029) with White’s test for heteroscedasticity again showing a significant result (ꭓ^2^(10) = 24.37, p = .007). Hierarchical regression testing was used to further explore the respective contribution of age as the stage 1 variable and group as the stage 2 variable on the MRBD. At stage one, age contributed significantly to the regression model, F(1,103) = 5.83, p = .018) and accounted for 5.4% of the variation in MRBD values. Introducing the group variable explained an additional 1.6% in MRBD value and this change in R^2^ was not significant (F(1,102) = 1.75, p = .188).

Post hoc tests indicate a difference between CT (-46.54 ± 2.89) and ES (-57.75 ± 3.24, q = .027) but not between CT and RO (-49.54 ± 3.51, q = .107), nor between the two patient groups (p = .524).

The right hemisphere MRBD also showed a significant effect of group F(2,101) = 3.48, p = .034. Hierarchical regression testing, with age as the stage 1 variable and group as the stage 2 variable, showed that at stage one, age contributed significantly to the regression model, F(1,104) = 6.74, p = .011) and accounted for 6.1% of the variation in MRBD values. Introducing the group variable explained an additional 2.3% in MRBD value and this change in R^2^ was not significant (F(1,103) = 2.58, p = .11). These results indicate that age accounts for the most variance between the groups.

Post hoc tests indicated a difference between CT (-45.35 ± 3.20) and ES (-57.11 ± 3.47, q = .030), but not between CT and RO (-49.12 ± 3.84, q = .430) nor between the two patient groups (q = .159).

These results indicate that age accounts for the most variance between the groups for both the left and right MRBD values.

*PMBR*

After removal of age as a covariate, there was still a significant main effect of group in the ANCOVA, F(2,99) = 11.9, p < .001. Post hoc tests indicate that the CT group (116.85 ± 8.93) were different to the ES group (56.92 ± 9.98, q < .001) and also the RO group (73.83 ± 11.12, q = .003). The patient groups were not significantly different (q = .230). In summary, there was no effect of covarying age on the pattern of results in the PMBR.

**Tests for relationship between symptom severity scales and patient group**

ANCOVAs with symptoms scores as the dependent variable and patient group (RO; ES) as the categorical predictor of interest, and with antipsychotic medication exposure, sex, site and age as covariates. These tests were also run without age as a covariate. Results were checked for homogeneity of variance using Levene’s test and heteroscedasticity using White’s test. Where Levene’s test is significant, we apply bootstrapping (10,000 samples; Bias-corrected and accelerated (BCa) standard errors and p-values).

*Total PANSS & effect of patient group*

There was no significant effect of patient group on Total PANSS with age included as a covariate: F(1,52) = .37, p = .55, nor without age included as a covariate: F(1,53) = .12, p = .73.

*Disorganization & effect of patient group*

There was no significant effect of patient group on Disorganization factor with age included as a covariate: F(1,52) = .17, p = .69, nor without age included as a covariate: F(1,53) = .22, p = .64.

*Impoverishment & effect of patient group*

There was no significant effect of patient group on Impoverishment factor with age included as a covariate: F(1,52) = .17, p = .69. In the test without age included as a covariate, Levene’s test was shown to be significant: Levene’s F(7,54) = 2.34, p = .037, and bootstrap-adjusted results show no significant effect of patient group: F(1,57) = 1.81, p = .18.

*Reality Distortion & effect of patient group*

In the test including age as a covariate, Levene’s test was significant: Levene’s F(7,54) = 2.37, p = .035, and bootstrap-adjusted results show no significant effect of group on Reality Distortion factor: F(1,56) = .143, p = .71. In the test without age as a covariate Levene’s test was significant: Levene’s F(7,54) = 2.24, p = .045, and bootstrap-adjusted results show no significant effect of group on Reality Distortion factor: F(1,57) = .544, p = .46.

**PMBR correlations with symptom severity**

A Pearson’s two-tailed partial correlation was performed for each patient group separately, controlling for age, site, sex and antipsychotic medication score.

*RO PMBR and total PANSS*

No significant correlation seen including age: R(22) = -.01, p = .962

No significant correlation seen without including age: R(23) = -.09, p = .669


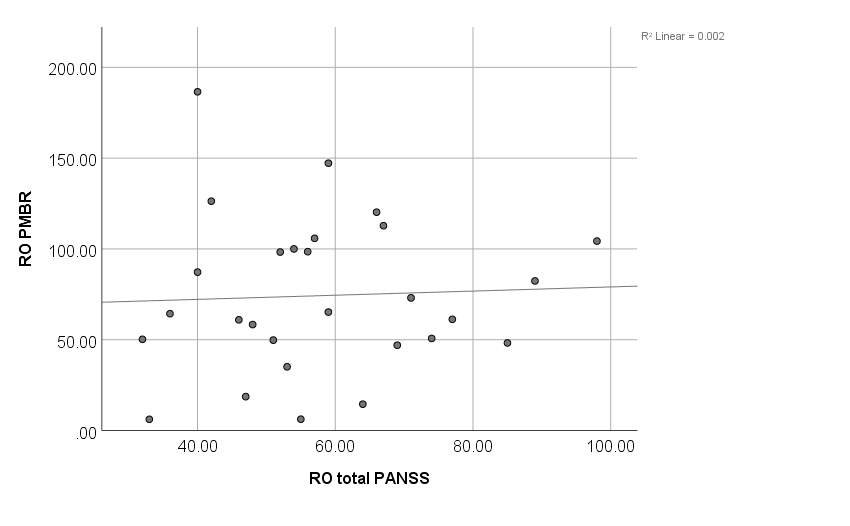


*RO PMBR and disorganization factor*

No significant correlation seen including age: R(22) = .061, p = .775

No significant correlation seen without including age: R(29) = .006, p = .978


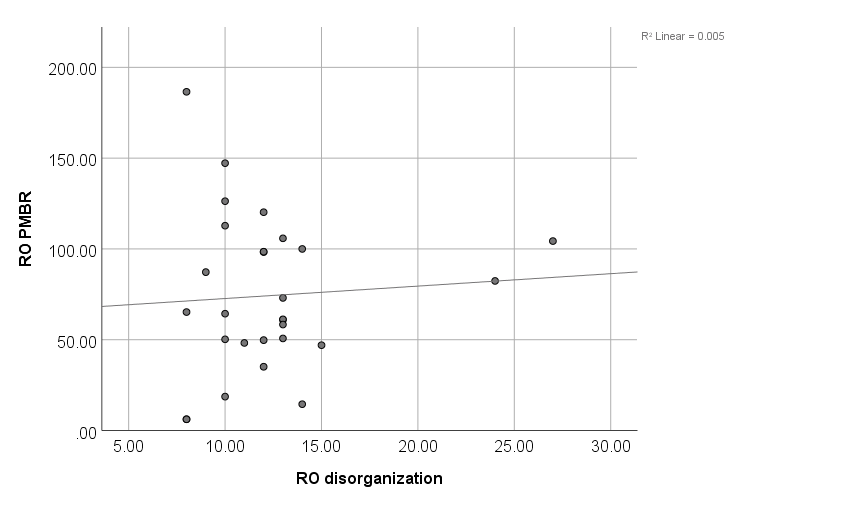


*RO PMBR and impoverishment factor*

No significant correlation seen including age: R(22) = .048, p = .981

No significant correlation seen without including age: R(23) = -0.94, p = .662


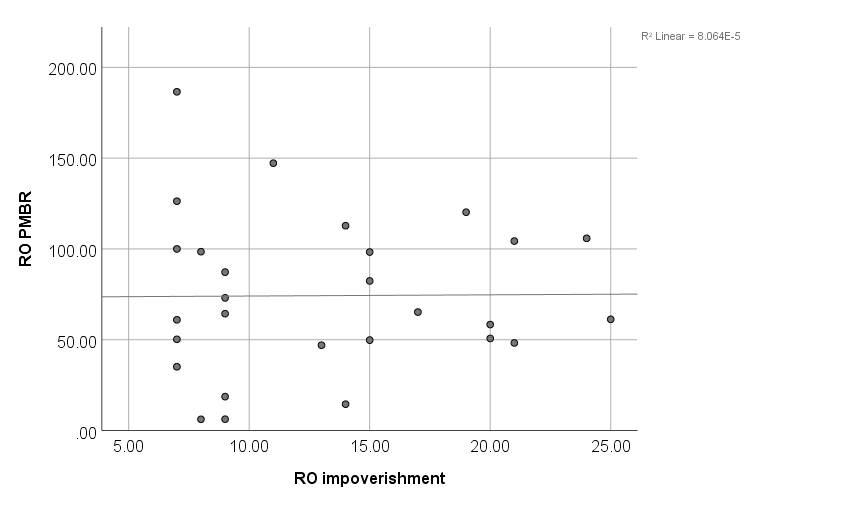


*RO PMBR and reality distortion factor*

No significant correlation seen including age: R(22) = -.173, p = .42

No significant correlation seen without including age: R(23) = -.216, p = .30


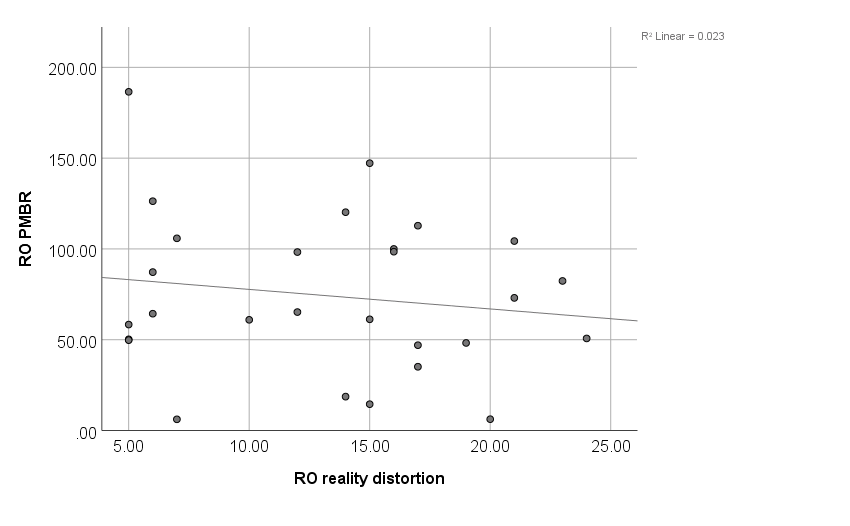


*ES PMBR and total PANSS*

No significant correlation seen including age: R(28) = -.336, p = .069

No significant correlation seen without including age: R(29) = -.336, p = .064


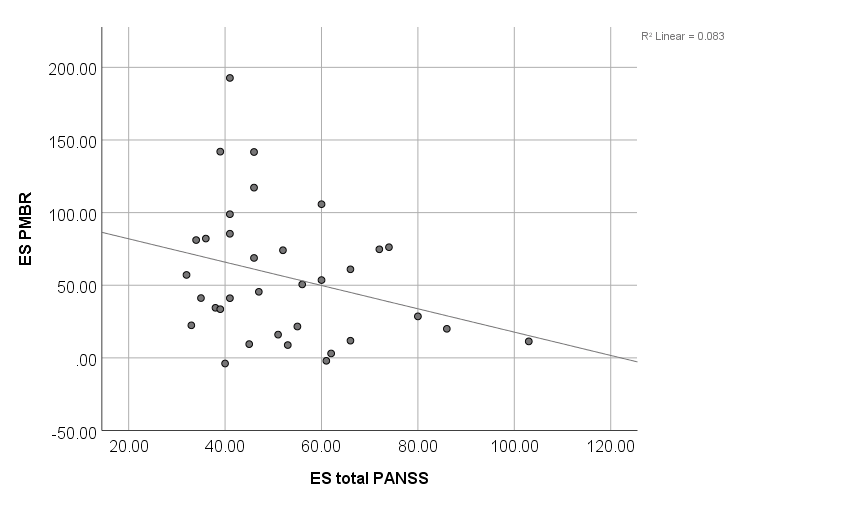


*ES PMBR and impoverishment factor*

No significant correlation seen including age: R(28) = -.132, p = .49

No significant correlation seen without including age: R(29) = -.132, p = .48

*
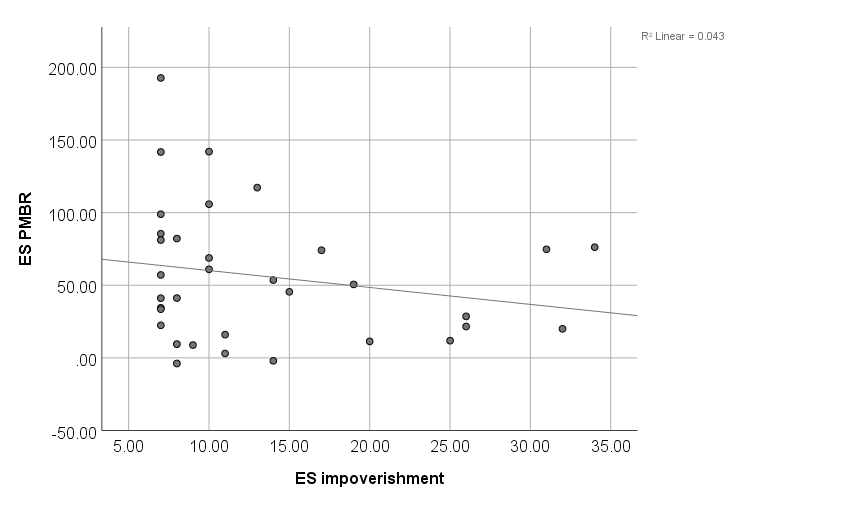
ES PMBR and reality distortion factor*

No significant correlation seen including age: R(28) = -.198, p = .301

No significant correlation seen without including age: R(29) = -.197, p = .290


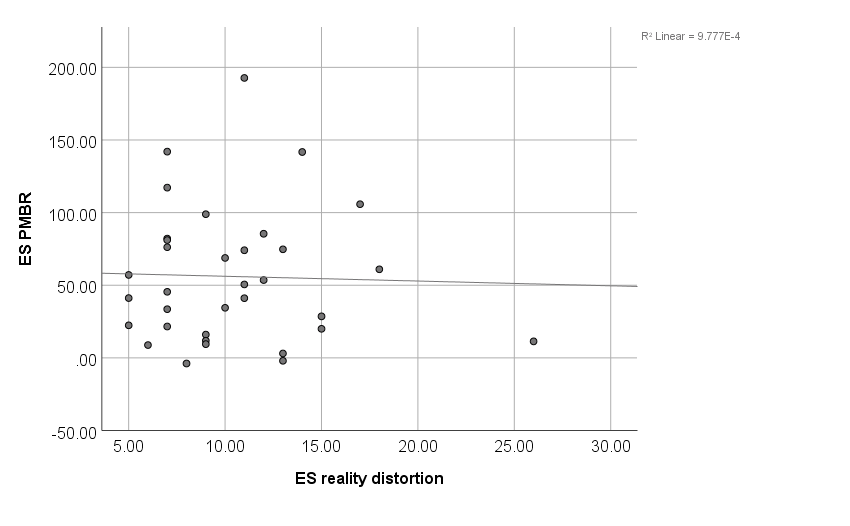


**Whole patient group correlations with PMBR**

*Total PANSS*

No significant correlation seen including age: R(56) = -.235, p = .075

No significant correlation seen without including age: R(57) = -.24, p = .068


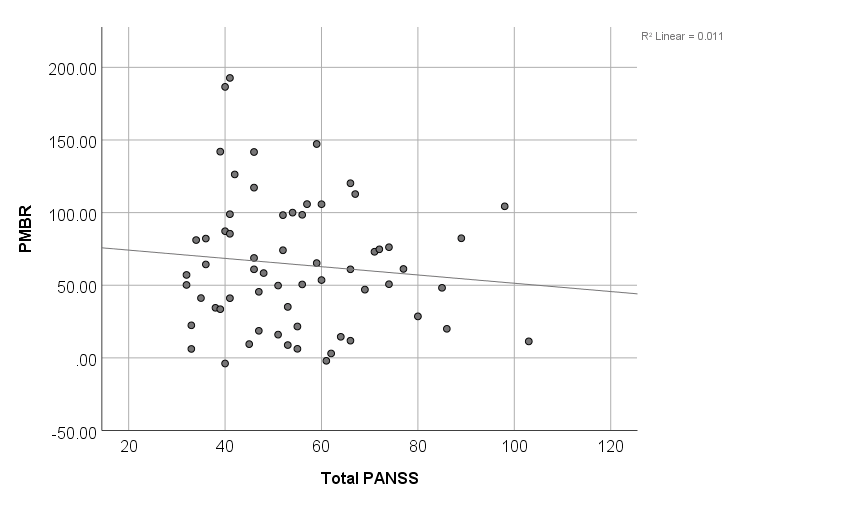


*Disorganization*

No significant correlation seen including age: R(56) = -.245, p = .064

No significant correlation seen without including age: R(57) = -.244, p = .063


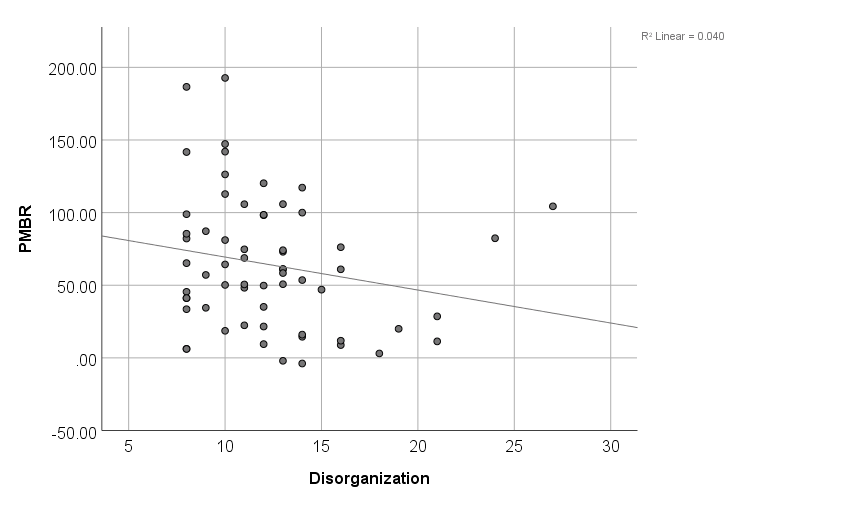


*Impoverishment*

No significant correlation seen including age: R(56) = -.143, p = .283

No significant correlation seen without including age: R(57) = -.149, p = .260


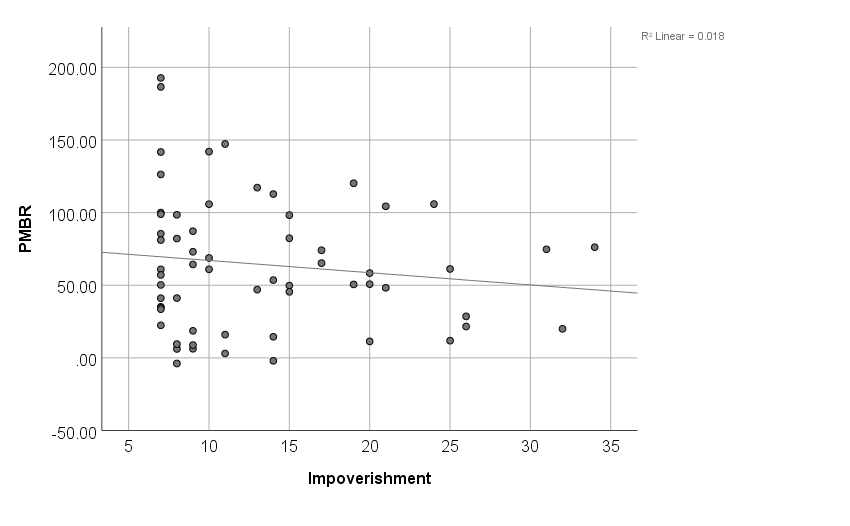


*Reality distortion*

No significant correlation seen including age: R(56) = -.172, p = .198

No significant correlation seen without including age: R(57) = -.178, p = .177


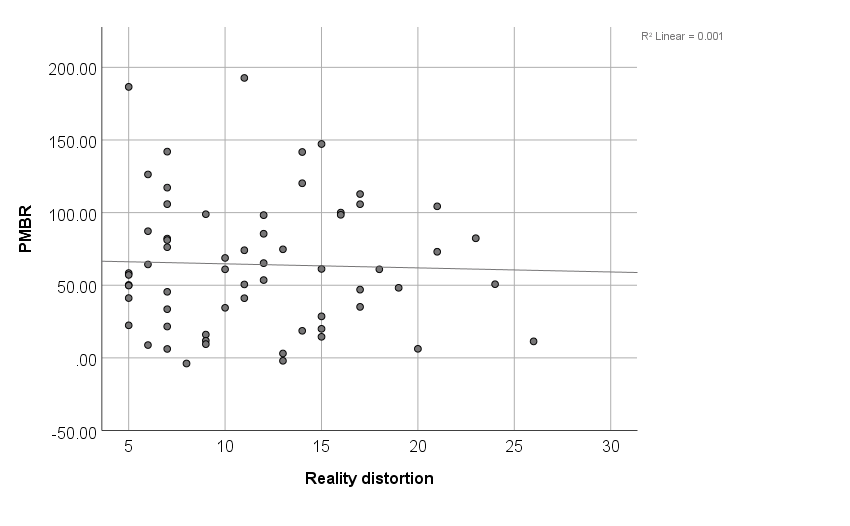


**HMM results – without including age as covariate**

*Burst amplitude*

After removal of age as a covariate there was a similar pattern of results for the main effect of group, F(2,74) = 6.59, p = .002. Further, the follow up tests showed a significant difference between the CT (4.77 ± .21) and ES group (3.61±.26, q = .003), but no difference between the CT and RO (4.19±.26, q = .101) nor the two patient groups (q = .105).

*Burst count*

After removal of age as a covariate, there was a significant main effect of group F(2,60) = 10.83, p < .001). Follow up tests indicated that there was a difference between CT (3.87 ± .25) and ES (2.44 ± .35, q = .002) and between CT and RO (2.35 ± .31, q < .001) but not between patient groups (q = .848).

*Burst duration*

After removal of age as a covariate, there was still a main effect of group (F(2,75) = 4.74, p = .01). Follow up tests indicated that there was a significant difference between the CT (3.58 ± .178) and ES (2.72 ± .23, q = .009), but not between CT and RO (3.26 ± .22, q = .251), nor between the two patient groups (q = .128).
